# The impact of different left heart decompression strategies and timing on VA-ECMO patients: a systematic review and network meta-analysis

**DOI:** 10.1097/JS9.0000000000002253

**Published:** 2025-01-30

**Authors:** Wei Wang, Rui Tang, Tianxiang Gu, Enyi Shi

**Affiliations:** Department of Cardiac Surgery, China Medical University, Shenyang, China

## Introduction

Veno-arterial extracorporeal membrane oxygenation (VA-ECMO) has emerged as a vital intervention for patients experiencing severe cardiac and respiratory failure, particularly those in cardiogenic shock. It serves as a conduit to recovery, heart transplantation, or long-term mechanical circulatory support. While VA-ECMO is a life-saving intervention, it does impose significant hemodynamic alterations, most notably an increase in left ventricular (LV) afterload. The retrograde blood flow from VA-ECMO into the aorta results in repeated reflux of blood into the LV, thereby increasing the left ventricular end-diastolic pressure (LVEDP). The pressurization and dilation result in heightened wall tension and increased myocardial oxygen demand, thereby exacerbating myocardial stress. In the event that the LV is unable to overcome this increased afterload, the resulting increase in LV volume and pressure can lead to LV dilation, decreased coronary perfusion pressure, and reduced stroke volume, thereby forming a vicious cycle. In the long term, this can cause a number of complications, including ventricular dilation, reduced coronary perfusion, arrhythmias, and pulmonary edema. Mortality rates in these cases range from 27% to 51%^[^1,2^]^. Left heart decompression (LHD) devices, including surgical and percutaneous approaches, can mitigate these adverse effects, thereby improving myocardial recovery and overall patient outcomes.

Surgical LHD is effective but invasive, carrying risks of infection and bleeding. In contrast, percutaneous techniques such as transseptal puncture, left atrium (LA) and pulmonary artery (PA) venting, and devices like the intra-aortic balloon pump (IABP) and Impella, provide alternative decompression options with different risk profiles. The choice of LHD approach is complex due to unique risks and benefits associated with each technique and limited comparative evidence. Moreover, the timing of LHD is critical; while early intervention may protect the LV, it can pose risks without clear indications^[^2^]^. Individual patient characteristics and clinical context often dictate the optimal timing and approach. Given these considerations, we conducted this systematic review and network meta-analysis (NMA) to provide a comprehensive evaluation of various LHD strategies and their timing, aiming to establish best practices for optimizing patient outcomes.

## Methods

This systematic review and meta-analysis followed PRISMA and AMSTAR guidelines and was registered on PROSPERO (https://www.crd.york.ac.uk/prospero). Inclusion criteria of relevant studies focused on VA-ECMO patients undergoing different LHD strategies. Two independent reviewers performed data extraction and bias assessment. Statistical analyses applied random effects models and NMA with surface under the cumulative ranking (SUCRA) to assess the efficacy and safety of LHD strategies. The risk of bias was assessed using The Cochrane Collaboration’s Tool for RCTs and the Newcastle-Ottawa Scale for retrospective studies. Statistical heterogeneity was assessed using the Cochran *Q* test and the *I^2^* test. If significant heterogeneity was observed (*I*^2^ > 50% or *P*[*Q*] < 0.05), a random effects model was used to calculate the pooled estimate. Otherwise, a fixed effects model was used. In instances of moderate to high heterogeneity among studies, sensitivity analyses were performed by sequentially excluding individual studies to assess their influence on the overall results. All analyses were conducted in R version 4.4.0, with significance set at *P* < 0.05.

## Results

Extensive searches in PubMed, Embase, Web of Science, and CNKI by May 2024, of which 34 studies were included in the NMA and six in the paired meta-analysis (Supplementary Figure 1, http://links.lww.com/JS9/D868). Studies examined various LHD strategies and outcomes, with high quality overall (Supplementary Figure 2, http://links.lww.com/JS9/D868).

**Figure 1.**
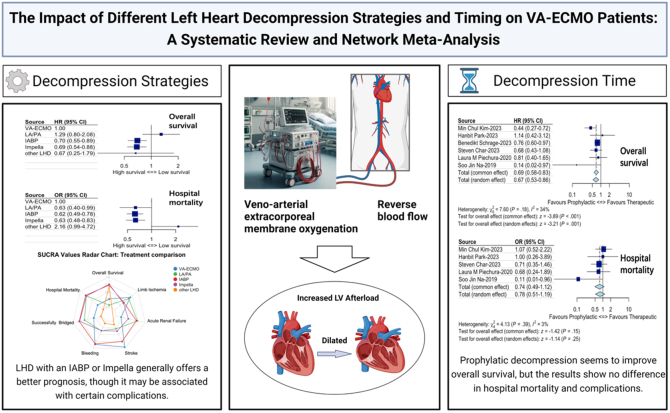
This study’s core content is illustrated as follows. The center depicts the effect of VA-ECMO on increased left ventricular afterload. The left side shows the primary results of the network meta-analysis and the SUCRA ranking of different decompression strategies (radar plot), while the right side displays the paired meta-analysis results regarding decompression timing. HR, hazard ratio; IABP, intra-aortic balloon pump; LA/PA, left atrium/pulmonary artery; LHD, left heart decompression; OR, odds ratio; SUCRA, surface under the cumulative ranking curve; VA-ECMO, veno-arterial extracorporeal membrane oxygenation.

The NMA included 9068 patients, categorized into five groups: VA-ECMO alone, VA-ECMO with LA/PA, IABP, Impella, and other LHD techniques (Supplementary Tables 1–3, http://links.lww.com/JS9/D869). Primary outcomes demonstrated that both IABP and Impella significantly improved survival and reduced hospital mortality compared to VA-ECMO alone (Supplementary Figures 3–4, http://links.lww.com/JS9/D868, *P* < 0.05). Secondary outcomes showed that stroke incidence was significantly lower in the IABP and VA-ECMO groups compared to Impella and LA/PA, while the bleeding risk with Impella was higher than with IABP and VA-ECMO (Supplementary Figure 5, http://links.lww.com/JS9/D868, *P* < 0.05). SUCRA values indicated that IABP and Impella ranked highest for survival, yet complications such as limb ischemia and acute renal failure remain significant concerns (Supplementary Figure 6, http://links.lww.com/JS9/D868). The result is shown in Figure 1.

The paired meta-analysis on decompression timing included six studies with a total of 850 patients, of which two^[^3,4^]^ were randomized controlled trials (RCTs). LHD was implemented prophylactically (within 12 hours of VA-ECMO) or therapeutically based on patient risk. Early LHD significantly improved survival (hazard ratio [HR]: 0.69, 95% confidence interval [CI]: 0.58–0.83, *P* < 0.01), but there was no significant difference in hospital mortality or adverse event rates between early and late LHD groups. The results are shown in Table 1.

**Table 1.** Meta-analysis results of primary and secondary outcomes for timing of LHD

	Studies	Effect measure	Model	Participants		Effect estimate	LCI	UCI	*P-*value
				Prophylactic	Therapeutic				
Primary outcomes									
Overall survival	6	Hazard ratio	Fixed effects	541	309	0.69	0.58	0.83	<0.01
Hospital mortality	5	Odds ratio	Fixed effects	105/231	95/198	0.74	0.49	1.12	0.15
Secondary outcomes									
Successfully bridged to transplant, LVAD, or myocardial recovery	5	Odds ratio	Fixed effects	72/231	55/198	1.34	0.81	2.21	0.26
Bleeding	5	Odds ratio	Random effects	241/483	112/251	0.72	0.36	1.46	0.36
Stroke	6	Odds ratio	Random effects	66/541	41/409	1.13	0.56	2.29	0.73
Infection	3	Odds ratio	Random effects	43/168	42/138	0.81	0.31	2.13	0.67
Acute renal failure	3	Odds ratio	Random effects	70/401	58/199	0.79	0.38	1.66	0.54
Limb ischemia surgery	4	Odds ratio	Fixed effects	10/139	17/150	0.62	0.27	1.41	0.25

LVAD, left ventricular assist devices; LHD, left heart decompression; LCI, lower confidence interval; UCI, upper confidence interval.

## Discussion

Despite the benefits, each LHD strategy carries unique risks. In this study, we explored the impact of various LHD strategies, including IABP and Impella, etc. on patients receiving VA-ECMO. The addition of these devices has demonstrated significant benefits in reducing mortality and improving survival rates, which validates their clinical relevance in managing the increased afterload and complications induced by VA-ECMO^[^5,6^]^. Both IABP and Impella showed clear advantages in overall survival and hospital mortality, with IABP further reducing stroke and bleeding risks compared to LA/PA and other LHD techniques. However, the elevated bleeding risk with Impella^[^7,8^]^, potentially linked to its larger vascular access requirements and high shear stress, raises concerns, as does the LA approach, which could induce additional trauma in non-surgical patients. The radar chart (SUCRA rank) clearly illustrates the complexities associated with each strategy, further underscoring the importance of evaluating the benefit-risk profile of each decompression method.

The timing of LHD also presents a number of clinical challenges. Although early prophylactic decompression may confer survival advantages by mitigating LV overload from the outset of VA-ECMO, our meta-analysis revealed no significant discrepancy in other outcomes between prophylactic and therapeutic decompression. The extant evidence indicates that early decompression, particularly within two hours, may diminish short-term mortality. However, outcomes appear to be device-dependent^[^9^]^. This may be attributed to the dynamic nature of patient hemodynamics during VA-ECMO support, where delayed LHD could exacerbate LV dilation, increase pulmonary congestion, and heighten the risk of secondary complications. To optimize the timing of LHD in clinical practice, it is essential to conduct continuous monitoring of hemodynamic parameters, including LVEDP, cardiac output, and PA pressures. By utilizing real-time physiological data, clinicians can ascertain the optimal window for intervention, thereby enabling the adaptation of decompression strategies to the specific characteristics of each patient. Furthermore, the potential variability in device efficacy highlights the necessity for patient-specific approaches and the careful selection of LHD devices to maximize therapeutic benefits. As non-randomized, observational data may conceal risks, particularly in patients nearing irreversible cardiopulmonary deterioration, these findings underscore the necessity for RCTs to more precisely define the optimal timing and choice of LHD device. Addressing these questions will be pivotal to developing standardized protocols that balance efficacy and safety, minimize adverse outcomes, and ultimately refine VA-ECMO therapy for high-risk patients.

## Limitations

The results of this meta-analysis should be interpreted with caution given several limitations. The study focused on survival and specific complications, with no data on hemodynamics, VA-ECMO duration, or lab indicators. Factors affecting VA-ECMO outcomes, such as hemodynamic variables, liver function, CNS dysfunction, and pre-ECMO intubation duration, were not consistently reported, complicating indirect comparisons. Although the overall quality of retrospective studies was acceptable, the lack of RCTs in the NMA necessitates careful interpretation. Variability in LHD methods and timing, along with potential biases in extracting HRs from survival curves, further emphasizes the need for high-quality RCTs to refine VA-ECMO protocols.

## Conclusion

To conclude, this meta-analysis indicates that IABP and Impella are the most effective LHD strategies for improving survival in VA-ECMO patients, with prophylactic decompression potentially enhancing outcomes. However, these findings require cautious interpretation due to significant limitations. Furthermore, high-quality RCTs are needed to confirm these results, optimize decompression strategies, and establish standardized protocols for VA-ECMO therapy.

## Data Availability

The datasets utilized in this study were obtained from journal publications and their supplementary appendices. All data generated or analyzed during this study are included in this published article.

## Research registration unique identifying number (UIN)

The protocol for this overview is registered on PROSPERO (CRD42024551712) and is accessible on the PROSPERO website.
